# Phthalates: the interplay between diet, obesity, diabetes, and gut microbiota

**DOI:** 10.3389/ftox.2026.1941846

**Published:** 2026-10-07

**Authors:** Luiza Czaczkowska, Natalia Radziwon, Klaudia Sikorska, Edyta Olkowska, Ewa Jabłońska, Wioletta Ratajczak-Wrona

**Affiliations:** Department of Immunology, Medical University of Bialystok, Bialystok, Poland

**Keywords:** diabetes, diet, EDCs, obesity, phthalates

## Abstract

Millions of tons of plastic are produced worldwide each year. Currently, it is almost impossible to avoid plastic and exposure to its effects. There are many chemical compounds that are used in its production. Some of them, classified as plasticizers, are phthalates, which are found in food packaging, toys, and many cosmetics. They give plastics durability, flexibility, and heat resistance. Humanity is exposed to these substances primarily through ingestion, i.e., processed food and its storage, and through inhalation of polluted air. Consequently, phthalates and their metabolites can be detected in the human body. Due to their ubiquity, they pose a global threat to human health. They contribute to overweight and obesity, insulin resistance, diabetes, and disorders of the nervous and endocrine systems. The purpose of this article was to review existing research on the impact of phthalates on diet, the development of obesity, and diabetes.

## Introduction

There is continuous development of civilization, and as a result, society is exposed to harmful substances. The environment plays a significant role in the development of obesity, diabetes, and other related diseases. Pollution, excessive consumption of processed foods, and exposure to substances that disrupt metabolism all contribute to the development of these diseases. As civilization develops, plastic production increases, leading to the widespread presence of microplastics (MPs), particles no larger than 5 mm in size, found in soil, water, air, and ultimately in food. Many compounds are used in the production of plastic, including phthalates, which are used as plasticizers. Their main function is to increase the flexibility, softness, and durability of polymeric materials, particularly polyvinyl chloride (PVC). These compounds belong to the group of endocrine-disrupting chemicals (EDCs). MPs are produced through the degradation of plastics and various other industrial processes. An increase in MPs in the environment has been observed to correlate with the incidence of metabolic syndrome (METS) and colon cancer. The most important metabolic disorders include obesity, hypertension, and dyslipidemia, which increase the incidence of cardiovascular disease and type 2 diabetes. EDCs also influence the composition of the gut microbiota, basal metabolic rate, and regulate appetite and satiety. Mechanisms such as chronic inflammation, oxidative stress, and endocrine dysfunction contribute to the development of these disorders. This may be caused, among others, by MP exposure, which contributes to excessive production of proinflammatory cytokines by activating signaling pathways, including NF-kB and MAPK (Kovacs et al., 2025; Albukhari, 2025; Sarni et al., 2021).

Direct contact with EDCs is unavoidable, as they can be found in several everyday products. Phthalates are one such group of compounds. These are esters and salts of phthalic acid used in the production of varnishes, adhesives, cosmetics, personal care products, perfumes, and packaging for food and drinks. Low molecular weight phthalates are often added to preserve odor, and high molecular weight phthalates are used in polyvinyl chloride (PVC). The avoidance of these substances, because of their wide use in various industries, is almost impossible (Ventrice et al., 2013; Palandri et al., 2026; Erdal et al., 2024). These compounds are based on phthalic acid anhydride and differ in the number of carbon atoms in their structure. The most common phthalates are bis(2-ethylhexyl) phthalate (DEHP), butyl benzyl phthalate (BBP), diisononyl phthalate (DiNP), diisodecyl phthalate (DiDP), diisobutyl phthalate (DiBP), and di-n-butyl phthalate (DnBP) (Erdal et al., 2024; Yumpu.com, 2003). Phthalates, being components of materials, cannot form covalent bonds and are considered a source of environmental contamination due to their ability to pass into water, food, and air (Lai et al., 2026; Chen et al., 2019; He et al., 2019). Though the half-life of these compounds is relatively short, repeated exposure results in a constant level of phthalates concentration in the blood or tissues. The concentration of phthalates often exceeds the permissible amount in the materials, despite the legal standards in force. The permitted level of DEHP, BBP, and DnBP in toys and care articles for infants and children is a maximum of 0.1% by weight of plasticizer-containing materials. The group of phthalates and their metabolites is shown in Figure 1 (Lai et al., 2026; Commission Regulation EU, 2018).

**FIGURE 1:**
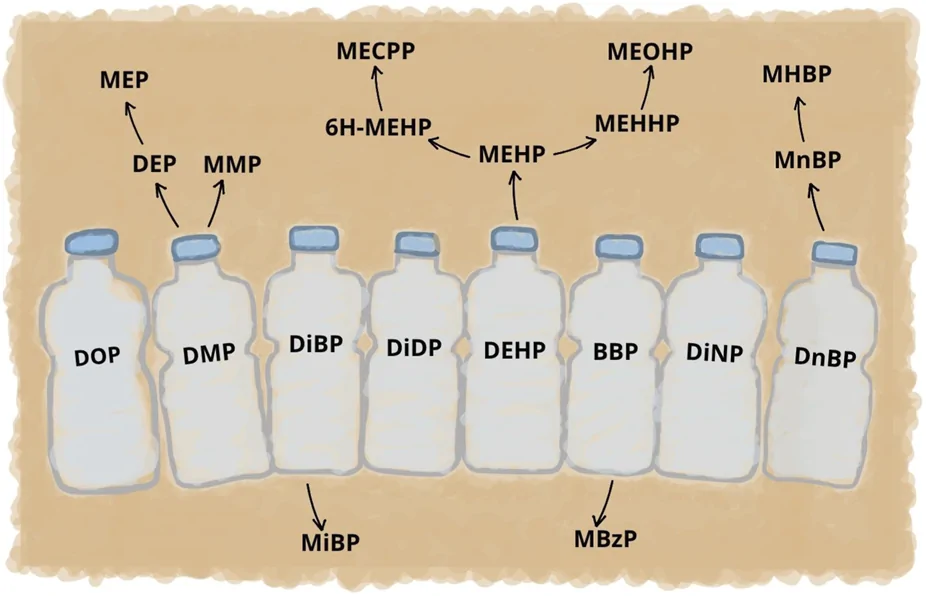
Group of phthalates and their metabolites (Lai et al., 2026; Commission Regulation EU, 2018).

DEHP, which is on the list of substances of very high concern (SVHCs), has been banned from use in the EU, except for certain industry applications. The maximum migration of this compound into food is limited to 1.5 mg/kg (Lai et al., 2026; Commission Regulation EU, 2018; Kourkopoulos et al., 2025; Regulation EC, 2025; Commission Regulation EU, 2025).

Phthalates may enter the human body through breathing, orally, dermally, and intravenously, as it is presented in Figure 2. Overall, human exposure to endocrine-disrupting chemicals such as plasticizers and per- and polyfluoroalkyl substances (PFAS) primarily occurs through diet and drinking water. However, due to their frequent contact with plastic, children are the most exposed to these compounds. Exposure to phthalates, including DEHP, may also occur during prenatal development. Furthermore, epidemiological and mechanistic evidence suggests a potential association between DEHP exposure and metabolic disturbances, including pathways relevant to metabolic syndrome (MetS), a cluster of interrelated cardiometabolic abnormalities including abdominal obesity, insulin resistance, hypertension, dyslipidemia, and impaired glucose metabolism (Chen et al., 2025; Jang et al., 2024; Kakavas et al., 2025).

**FIGURE 2:**
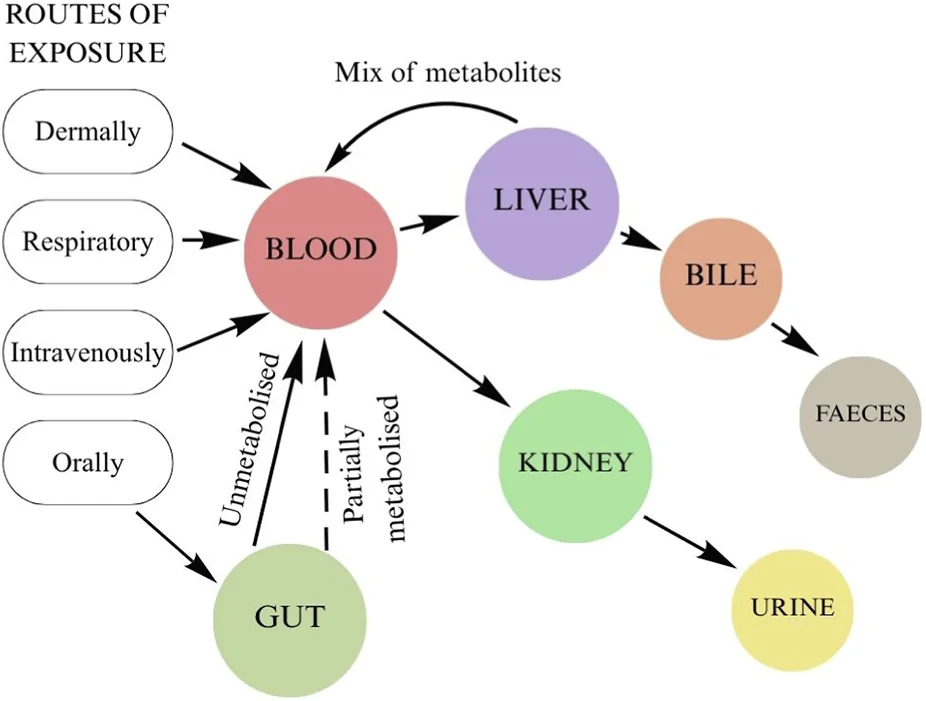
Routes of exposure to phthalates (Lai et al., 2026; Commission Regulation EU, 2018; Kourkopoulos et al., 2025; Regulation EC, 2025; Commission Regulation EU, 2025).

After phthalates are ingested orally, they undergo hydrolysis in the intestines under the influence of esterase and lipase. This results in the formation of low molecular weight phthalate metabolites monoesters such as monoethyl phthalate (MEP), mono-n-butyl phthalate (MnBP), and mono-isobutyl phthalate (MiBP). Dimethyl phthalate (DMP) produces monomethyl phthalate (MMP), and diethyl phthalate (DEP) is converted to (MEP). Before phthalates are conjugated with glucuronic or sulfuric acid, they undergo phase I oxidative metabolism and are excreted in the urine in the range from 6 to 24 h of exposure (Jang et al., 2024; Dai et al., 2025; Mínguez-Alarcón et al., 2025).

It is worth noting that phthalates are generally considered non-persistent compounds in the human body and have relatively short biological half-lives. Following exposure, they are rapidly metabolized, and their metabolites are predominantly excreted in urine. Consequently, urinary phthalate metabolites are widely used as biomarkers for assessing recent phthalate exposure (Jang et al., 2024; Braun et al., 2012; Domínguez-Romero and Scheringer, 2019). Although serum has been used as a biological matrix in some biomonitoring studies, concentrations of most phthalate metabolites are generally considerably lower in serum than in urine. This difference reflects the rapid metabolism of phthalates and the efficient clearance and urinary excretion of their metabolites, making urine the preferred matrix for assessing exposure to these non-persistent compounds (Caporossi et al., 2022; Liu et al., 2023; Gonkowski et al., 2024).

Some phthalates are also often added to cosmetics, where they act as fragrance stabilizers. MEP, being the biomarker used to assess phthalate exposure, was measured, and its concentrations were found higher among women comapared with men. This may be due to the fact that women use more cosmetic products. Unfortunately, observing this phenomenon in pregnant women is not reassuring. The same metabolite was also detected in a study where men who used either cologne or aftershave within the 48 h before urine collection had higher levels of MEP (Foley et al., 2025; Rosen et al., 2024).

## Materials and methods

The study conducted research on the PubMed and Google Scholar Platforms. The articles used in our study were meta-analyses, cohort studies, epidemiological studies, integrated bioinformatic analysis, research articles, *in vitro* studies, and case–control studies with relevance for phthalates in diet, obesity, and diabetes. The legal acts and regulations in this article were researched on a website dedicated to European Union legal regulations. References included publications from 2003 to 2026, with a significant portion of the scientific literature published in recent years. Articles were excluded if they did not provide sufficient data to support their conclusions or did not contribute relevant additional information to the scope of this review.

## Phthalates and diet

Leading a healthy lifestyle relies primarily on maintaining a balanced diet, which reduces the risk of developing various diseases and is crucial during their treatment. Excessive food consumption will lead to problems with nutrient storage. Excessive absorption and storage of nutrients can lead to a number of metabolic diseases, such as obesity and diabetes. Macro- and micronutrients supplied to the body with food are responsible for regulating vital and metabolic processes at the level of cells, tissues, organs, and systems. Vitamins and minerals belonging to the micronutrient group play an important role in supporting the immune system (Oliveira Moura et al., 2025).

Additionally, toxic compounds that are detected in food could also have potentially harmful effects in the disruption of many key biochemical transformations. Thus, the quality of food can affect both physical and mental health. Despite a complete ban, phthalates are still found in food products. Since many manufacturers still place and sell their products in plastic packaging, it may pose a serious threat and a significant source of phthalate exposure due to the migration of these compounds from packaging materials into food (Xu et al., 2025; Bartolomé et al., 2026).

Based on COMMISSION REGULATION (EU) No 10/2011 of 14 January 2011 on plastic materials and articles intended to come into contact with food, including guidelines for phthalates, were established. The European Union List of authorized substances in the plastics production process has also been adopted (Lai et al., 2026; Kourkopoulos et al., 2025; Regulation EC, 2025; Commission Regulation EU, 2025).

When following a proper diet, adequate hydration of the body is important. Water makes up about 60% of an adult’s body weight, and in newborns, it makes up more than 80%. Maintaining body temperature at an appropriate level, transporting nutrients, and participating in almost all of the biochemical transformations taking place in the body are some of the main functions of water. The World Health Organization (WHO) made Food-Based Dietary Guidelines recommendations for water for each EU country, Iceland, Norway, Switzerland, and the United Kingdom. Most of them have recommendations to drink 1.5–2.0 L of water per day. However, it is important to note that water can also be consumed with food. Because of the widespread use of water sold in bottles made of plastic, researchers investigated how storage can affect the level of phthalates in the composition of water. Some possible sources of these compounds in bottled water can be the technological processes used during production, chemicals used to treat the water, and previously recycled PET packaging, which is used for remanufacturing of bottles (Food-Based Dietary Guidelines, 2025; Goessens et al., 2026; Shinohara et al., 2026).

There is a need to verify whether the compounds used in the production of bottles and other plastics in recycled plastics do not exceed legal limits. It is especially important in light of the promotion of recycling and the global trend of reducing plastic waste. Plasticizers and flame retardants together account for approximately 50% of the weight of all plastic additives. The study by Gerassimidou et al. showed that recycled plastics release phthalates at levels that exceed regulatory migration limits. Shinohara et al. detected many phthalates in plastic pellets made in Japan, where DEHP was found the most frequently. DiDP has not been detected, probably because it is rarely used in this country. In contrast, in the EU and U.S., DiBP and DnBP were detected in 45% and 64% of analyzed pellet samples (Shinohara et al., 2026; Plastics Europe, 2026; Gerassimidou et al., 2023).

In China, Wang et al. analyzed tap water, barrel water, and bottled water for phthalates and their ability to migrate from bottles and barrels. It was found that the concentration of DEHP was the highest, while BBP and DiBP were slightly lower in concentration. The total concentration of phthalates was 2.409 ± 0.391 μg/L in tap water, 1.495 ± 0.213 μg/L in barreled water, and 1.963 ± 0.160 μg/L in bottled water. This study also proved that the concentration of plasticizers increases in direct proportion to the temperature and length of storage of the barrel or bottled water (Wang et al., 2021). In another study done by Bartolomé et al., three hazardous chemicals, which belonged to the phthalate group, associated with plastic packaging were determined. Benzylbutyl and diisobutyl phthalates were found in potato microwavable plastic packaging, and diisodecyl phthalate in the oven bag for meat (Bartolomé et al., 2026).

It is known that cold-pressed oils are a source of unsaturated omega-3 and omega-6 fatty acids. Since these fats are an essential component of the daily diet, they must be supplied with food, as the body cannot produce them. These acids are characterized by many beneficial properties. They are responsible for regulating blood pressure, lowering triglycerides, protecting against heart attacks and other coronary artery diseases, supporting brain function, and improving skin conditions. However, the presence of phthalates in these oils also contributes to disorders of the body’s homeostasis (Goessens et al., 2026).

In Turkey, a total of 30 different cold-pressed oils were tested. DEHP was detected in 18 of the 30 tested samples, at concentrations ranging from 0.56 to 92.12 mg/kg. The next most common plasticizer was DiBP, which was identified in six samples at concentrations ranging from 0.10 to 51.63 mg/kg. The presence of these phthalates in the tested oils may be due to their migration from the polymeric materials in the machinery, tools, and containers used in the production and storage of cold-pressed oils (Kiralan et al., 2020).

Pregnant women, infants, and children are particularly vulnerable to toxic compounds. Pregnant women must pay special attention to supplementing essential nutrients for the proper development of their children. These nutrients include folic acid, iron, calcium, and omega-3 fatty acids. Indicators such as the Diet Quality Score [Healthy Eating Index (HEI)] and the Diet Quality Index (DQI) reflect the adequacy of diets to general recommendations for specific populations. Adhering to an appropriate diet during pregnancy can reduce the risk of gestational diabetes, gestational hypertension, preeclampsia, and preterm birth, and increase the chances of normal childhood growth and obesity risk for the child (Mitran et al., 2024).

Rahimlou et al., in their systematic review study, have shown that phthalates interfere with lipid synthesis, transport, and metabolism, as well as modulate lipid-related gene expression. Phthalate esters (PAEs) can penetrate the placenta and affect the growth and development of newborns. Exposure to phthalates has been linked to lipid metabolism and dyslipidemia in both animal and human studies (Rahimlou et al., 2024).

Another concern about the source of PAEs in food is related to their presence in the environment. They reach water and soil through dry sedimentation and rainwater leaching. The U.S. Environmental Protection Agency (EPA) has listed six common PAEs: BBP, DnBP, DEHP, DEP, DMP, and dioctyl phthalate (DOP), as priority pollutants with potential impact on other organisms. Hong et al. examined total PAEs concentrations in the Yangtze River Delta region. DnBP was the predominant PAE in water, while DEHP dominated in soil and sediments. Food production is closely linked to the health of the ecosystem. This is particularly concerning, as there is a risk that vegetables, fruits, and meat produced in this region may contain these compounds (Xu et al., 2025; Hong et al., 2025).

Through their impact on the environment, phthalates may also have a negative effect on the animals living there. However, the exposure of wild animals to phthalates remains poorly understood. Gonkowski et al. conducted a study on the concentration levels of phthalate metabolites in wild boar hair samples. To date, studies have primarily been conducted on fish and marine mammals, with a few studies conducted on terrestrial animals. Because wild boars live in close proximity to human habitation, they are also exposed to various pollutants. The authors detected high concentrations of the metabolites of DEHP, mono(2-ethylhexyl) phthalate (MEHP), and the metabolite of DMP, MMP, in samples from both industrialized and agricultural areas. The presence of these metabolites is associated with the previously described phenomenon of these substances penetrating groundwater and soil (Gonkowski et al., 2024).

Another study by Gonkowski et al. examined the long-term effects of phthalate exposure by examining the concentrations of MMP, MEP, MiBP, MnBP, monobenzyl phthalate (MBzP), monocyclohexyl phthalate (MCHP), and MEHP in hair samples using liquid chromatography with mass spectrometry (LC-MS/MS) analysis. Of these metabolites, only MCHP was not detected in isolated samples. MEHP and MiBP were present in all tested samples. The authors also demonstrated that exposure to these compounds is higher in indoor cats than in outdoor cats. This suggests that indoor cats are more exposed to these compounds. Phthalate concentrations in cats should be compared with those in the hair of their owners. This would allow for a comparison of their exposure to these compounds (Gonkowski et al., 2025).

To investigate phthalate toxicity while reducing reliance on animal experiments, *in vitro* approaches, particularly cell culture models, are increasingly used. Tekin et al. cultured parenchymal cells from the right and left testicles of three dogs after routine castration in Turkey. They examined phthalate exposure for its toxicity on male fertility. DMP showed the lowest toxicity among the compounds tested, while DEHP showed the highest. In the study by Yurdakok-Dikmen et al., the authors also tested levels of BBP, DnBP, DiBP, DiNP, DEHP, DEP, and DMP on bovine primary testicular and epididymal cells *in vitro*. This shows that not only humans are exposed to phthalates in food and water, but also pets and cattle (Tekin et al., 2020; Yurdakok-Dikmen et al., 2019).

## Phthalates and obesity

Obesity is a serious medical condition that elevates rates of chronic diseases such as diabetes, cardiovascular diseases, cancers, hypertension, strokes, and asthma. In severe cases, it can lead to premature death. It is considered the most common chronic disease worldwide. It is alarming that the percentage of obese people in society increases year by year. This disease is diagnosed based on a Body Mass Index (BMI) over 30 kg/m^2^ and can be divided into three stages. Group 1 includes a BMI <35 kg/m^2^, group 2 a BMI <40 kg/m^2^, and group 3 a BMI >40 kg/m^2^. There are scientific reports suggesting that prenatal exposure to phthalates may contribute to increased BMI and height. This is explained by the impact of these obesogens on lipid and carbohydrate metabolism and the induction of oxidative stress. As reported by Dalamaga et al., obesity also has a strong genetic basis. Family and twin studies have shown heritability rates from 40% to 70% for BMI. Over 95% of obese individuals have polygenic obesity, the etiology of which remains unclear (Jaskulak et al., 2025; Sol et al., 2025; The Role of Endocrine Disruptors in Childhood Obesity, 2020; Dalamaga et al., 2024).

As it is presented in Figure 3 *,* the factors contributing to the increase in weight, fat tissue, and thus obesity include: sedentary lifestyle, limited exercise, excessive consumption of highly processed foods, consuming much more food than the body needs for energy, and genetic predisposition. The imbalance between excess energy intake and reduced energy expenditure results in development of pro-inflammatory conditions. The “exposome” theory, which points to external effects in exposure, is worth considering. The external factors are also considered to be endocrine-disrupting chemicals. They are easily released into the environment because these compounds cannot covalently bind to plastics. EDCs have the ability to disrupt hormonal balance by binding as agonists or antagonists to hormonal receptors, impairing the normal biosynthesis, secretion, and metabolism of hormones (La Merrill et al., 2020). Additionally, since their chemical structure is similar to that of endogenous hormones, they can bind to estrogen, progesterone, androgen, and aryl hydrocarbon receptors (AhR), thus influencing the hormonal balance and, consequently, the metabolism of fats and carbohydrates (The Role of Endocrine Disruptors in Childhood Obesity; Akritidis et al., 2025; Giulivo et al., 2016).

**FIGURE 3:**
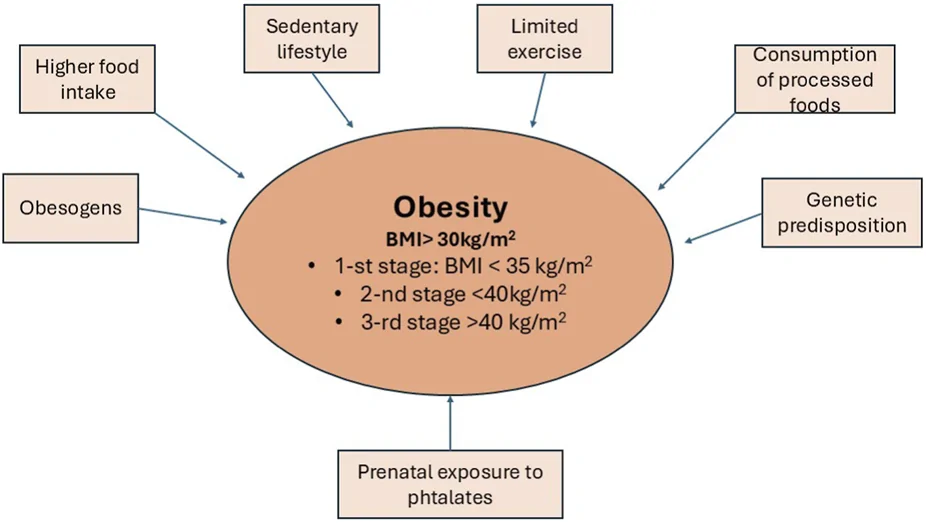
Obesity (The role of endocrine disruptors in childhood obesity; Akritidis et al., 2025; Giulivo et al., 2016).

The disruption of hormone homeostasis can lead to a number of diseases in humans, including overweight and obesity. EDCs that promote weight gain are referred to as “obesogens”, which are characterized by their ability to promote adipogenesis and lipid accumulation. The effects of obesogens on the human body include increased appetite, thus regulating metabolic homeostasis and disrupting adipose tissue differentiation. Two pathways are responsible for regulating food intake: the homeostatic and hedonic circuits. The homeostatic pathway regulates food intake according to the body’s needs, while the hedonic pathway drives appetite and food intake, not based on energy needs, but rather to satisfy thirst and provide the body with pleasure. Both pathways work together, and any disruption in their functioning causes adverse effects on the body. Disruptions in the neurotransmitter signalling involved in regulating feelings of satiety, hunger and pleasure can lead to obesity. Obesogens disrupt this balance. The consumption of contaminated food represents the major route of human exposure to obesogens. Consuming highly processed foods also contributes to this risk. Certain medications can also influence the development of obesity (Yilmaz et al., 2025; Kladnicka et al., 2022).

Adipose tissue, which is composed of adipocytes, is responsible for the storage of energy and its metabolism. There are three recognized adipose depots in the human body: the white, the brown, and the beige. Excessive formation of adipocytes and the adipokines they produce can lead to stimulation of hunger and obesity. The growing body of evidence supports the idea of the possible impact of obesogens on adipogenesis, fat accumulation, appetite control, and thus obesity (Kladnicka et al., 2022).

Epigenetic changes in the mechanisms modulating gene expression in adipose tissue may be closely linked to the action of phthalates in early development, childhood, and predisposition to obesity. These changes may influence DNA methylation, the activity of DNA methyltransferases and their cofactors, and modulate locus-specific epigenetic patterns. Dalamaga et al. also mention that BBP may induce differentiation of 3T3-L1 cells and C3H10T1/2 stem cell lines, with increased expression of miR-34a-5p, which is involved in adipogenesis, obesity, and the epigenetic regulation of insulin signaling. MEHP at a concentration of 10 µM activates undifferentiated pre-adipocytes and, through increased glucose transporter expression, leads to lipogenesis. Lipid accumulation is induced by inhibition of Janus kinase (JAK)2/signal transducer and activator of transcription (STAT)5 signaling. In mature adipocytes, this can lead to insulin resistance and increased adipokine production (Dalamaga et al., 2024).

There are several mechanisms through which phthalates can induce obesity, such as the activation of the peroxisome proliferator-activated receptor γ (PPARγ), increased appetite, disruption of lipid metabolic pathways, and induction of adipogenesis and fat storage. Among these mechanisms, activation of PPARγ appears to be particularly important. This receptor plays a crucial role in adipocyte differentiation and regulates lipid metabolism and lipase activity. Studies conducted by Kandel et al. have shown that PPARγ influences insulin metabolism and glucose catabolism (Nameni and Kasiri, 2025).

Peroxisome proliferator-activated receptors are classified as transcription factors and are responsible for regulating the expression of certain genes. They belong to the nuclear receptor family, which, besides PPARγ, includes PPARα and PPARβ. They are activated by endogenous ligands, including fatty acids and their metabolites. Upon ligand binding, PPARγ forms heterodimers with retinoid X receptors (RXRs). The PPARγ–RXR heterodimer binds to specific peroxisome proliferator response elements in DNA and regulates the transcription of target genes. Its high expression in adipose tissue reflects its central role in adipocyte differentiation, lipid storage, and energy homeostasis. Furthermore, mutations in the PPARγ gene have been implicated in the development of metabolic disorders in humans, notably lipodystrophy, highlighting the receptor’s critical role in adipose tissue function and energy balance (PMC, 2026).

The occurrence of metabolic syndrome and other metabolic diseases has been associated with factors, such as diet, lifestyle, genetics, and aging. However, as research and evidence accumulate, environmental factors, including EDCs, are being implicated in the development of metabolic diseases. This led to the formation of the “metabolism-disrupting chemicals (MDCs) hypothesis,” which proposes that substances present in the environment may lead to dysregulation of metabolic processes, contributing to the development of metabolic disorders such as diabetes and obesity. MDCs primarily affect the liver and adipose tissue. The liver is a key organ for metabolizing glucose and fatty acids. Hepatocytes have the ability to detect and respond to signals sent from, among others, adipose tissue, the intestines, the thyroid gland, and specific liver signaling molecules. Another important function of this organ is energy metabolism, in which the liver responds to insulin and blood glucose levels. This initiates the process of glycogenesis, which involves the synthesis of glycogen from glucose, whereas glucose can be metabolized through glycolysis to pyruvate. Pyruvate can be used as an energy source or to produce fatty acids through *de novo* lipogenesis. Experimental evidence indicates that some phthalates, particularly di(2-ethylhexyl) phthalate and its primary metabolite mono(2-ethylhexyl) phthalate, can interfere with hepatic lipid homeostasis. These compounds have been shown to alter lipid metabolism, promote lipid accumulation, and induce oxidative stress involving PPARα and the SREBP-1c signaling pathway in human HepG2 liver cells (Fritsche et al., 2023). Pregnancy and the associated biological and lifestyle changes constitute a key event in a woman’s life, which predisposes her to subsequent weight gain, metabolic disorders, and the development of chronic diseases. Traditionally, pregnancy is regarded as a period that determines the health and development of the child. However, the same physiological changes that influence the child’s development also affect the mother and may play a role in shaping her long-term health. Women with high gestational weight gain are more likely to develop gestational diabetes mellitus, gestational hypertension, and postpartum weight retention, all of which are risk factors for poor cardiometabolic health outcomes, including type 2 diabetes, cardiovascular diseases, and obesity. Although diet and exercise are strongly associated with weight gain both during and after pregnancy, there is growing evidence that environmental exposures, such as endocrine-disrupting chemicals, may have influential roles (Akritidis et al., 2025; Zukin et al., 2021).

In a study by Deierlein et al., attempts were made to find a relationship between prenatal exposure to phthalates, weight during pregnancy and weight up to 7 years after delivery. Because a mother’s lifestyle and exposure to various compounds significantly impact child development, they may also contribute to other long-term outcomes. Women with abdominal obesity or who are overweight are at risk for a number of cardiovascular diseases. The authors demonstrated a positive correlation between increased urinary metabolite concentrations and weight gain. Other studies have also shown that higher urinary metabolite concentrations in the women studied were associated with greater early gestational weight gain (Deierlein et al., 2022).

Studies on the effects of EDCs on the development of obesity are being conducted not only in humans but also in animals. An example is an experiment conducted in 2021, in which researchers confirmed the effect of bis(2-ethylhexyl)-tetrabromophthalate (TBPH) on the development of obesity in female freshwater fish, such as zebrafish. For 6 weeks, the fish were fed a normal-fat or high-fat diet and exposed to TBPH. The results obtained from this study show that exposure to TBPH has a significant impact on adipocyte diameter, body weight gain, and subcutaneous fat accumulation. These effects were exacerbated by the high-fat diet. This may be due to the transcription of adipokines, i.e., leptin and adiponectin, which are associated with the brain-gut axis, and changes in the composition of the gut microbiome, which contribute to obesity. Additionally, increased expression of PPARγ was observed in the intestine. However, the study authors suggest conducting further research on the mechanism of action of TBPH, its toxicity, and its impact on the microbiota (Zhou et al., 2021).

## Phthalates and diabetes

According to the Polish Diabetes Association (Polskie Towarzystwo Diabetologiczne; PTD), diabetes is a group of metabolic diseases characterized by hyperglycemia resulting from defects in insulin secretion and/or action. Chronic hyperglycemia is associated with damage, dysfunction, and failure of various organs, especially the eyes, kidneys, nerves, heart, and blood vessels. These include type 1 diabetes, type 2 diabetes, genetic defects in β-cell function, genetic defects in insulin action, diseases of the exocrine pancreas, endocrinopathies, drug- and chemical-induced diabetes, infections, rare forms of immune-mediated diabetes, other genetically determined syndromes associated with diabetes, and gestational diabetes. Some factors that predispose to the development of diabetes are poor eating habits, overweight, obesity, stress, low physical activity, and the action of harmful compounds, such as phthalates. Some of the risks were illustrated in Figure 4 (26th Annual Meeting of Diabetes Poland Cracow, 2025).

**FIGURE 4:**
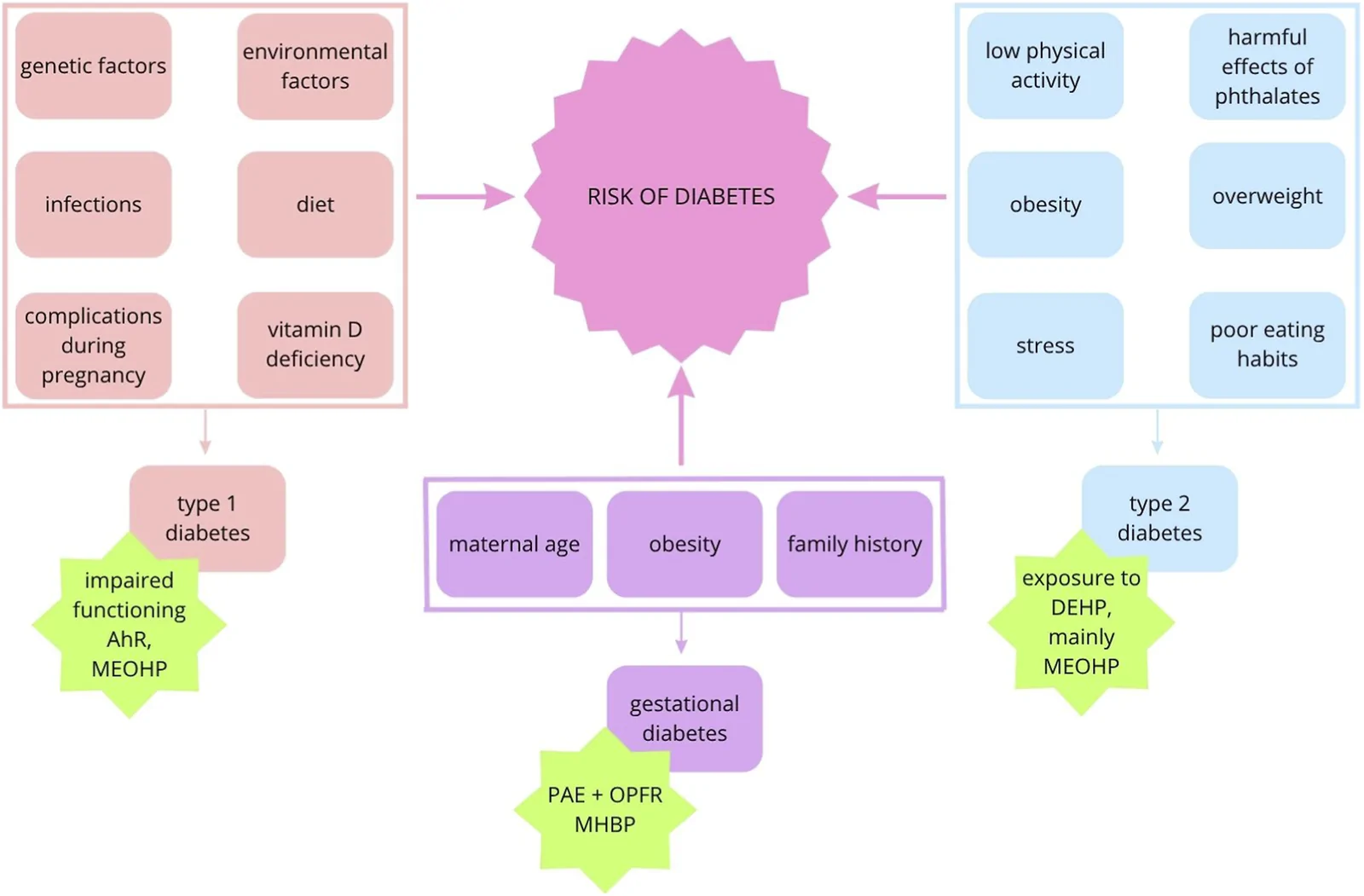
Risk factors for diabetes and the possible influence of phthalates on its development (26th Annual Meeting of Diabetes Poland Cracow, 2025).

The current classification of diabetes does not distinguish LADA (Latent autoimmune diabetes of adults), which is considered one of the clinical forms of type 1 diabetes. Monogenic diabetes includes MODY (maturity-onset diabetes of the young), mitochondrial diabetes, and permanent neonatal diabetes. Obesity is a predisposing factor to the development of type 2 diabetes. It is caused by the progressive loss of the ability of pancreatic β cells to secrete insulin properly, accompanied by insulin resistance (Zukin et al., 2021; 26th Annual Meeting of Diabetes Poland Cracow, 2025).

Type 1 diabetes (T1DM) is an autoimmune disease increasingly common in children and adolescents. In 2024, over nine million people with T1DM were registered, nearly two million of whom were under 20 years of age (International Diabetes Federation, 2026). The characteristic symptom of this disease is insulin deficiency or lack of secretion caused by the destruction of pancreatic β-cells by the immune system. Factors increasing the risk of T1DM include genetic and environmental factors, complications during pregnancy, diet, infections, and vitamin D deficiency. In general, EDCs can impair β-cell function, initiating autoimmunity, but there is insufficient research on the effect of EDCs on the development of T1DM. It is possible that the AhR, acting as a transcription factor, plays a key role in this mechanism. AhR regulates important processes in cells, primarily the immune response and the metabolism of foreign substances. In early life, EDCs can act as agonists or antagonists of the AhR, leading to disruption of cellular and molecular pathways and the endocrine system, and can also alter gene transcription. AhR is present in immune cells, such as T lymphocytes, which have the ability to destroy pancreatic β-cells. Some reports indicate that, depending on the cell type in which the receptor is activated, immunosuppression and changes in the immune response may occur during the development of T1DM. This effect points to the potential impact of AhR on the development of T1DM. Although numerous studies indicate the influence of EDCs on the occurrence of, for example, pancreatic diseases, evidence for the role of AhR in the pathogenesis of this disease is still lacking (Keskesiadou et al., 2024; Kim, 2024).

According to the latest WHO data from 2025, approximately 17% of women worldwide will experience gestational diabetes, which is characterized by abnormal carbohydrate metabolism and is first diagnosed during pregnancy. The key mechanisms responsible for the development of this type of diabetes are insulin resistance and glucose intolerance. It is primarily caused by impaired pancreatic β-cell function. Gestational diabetes (GDM) poses a health risk to the mother, increasing the risk of hypertension or postpartum type 2 diabetes, and to the child, who will be at increased risk for obesity, type 2 diabetes, metabolic syndrome, and cardiovascular disease. Traditional predisposing factors for GDM include maternal age, obesity, and family history. However, in the majority of the population without these factors, the risk of diabetes is caused by exposure to environmental pollutants (Mariana and Cairrao, 2023).

This is confirmed by a study conducted by a group of scientists from China in 2024, who investigated the relationship between organophosphate flame retardants (OPFRs) and PAEs with GDM. The study included 100 control subjects and 65 women with GDM, whose urine was tested for these substances. The results showed higher concentrations of phthalates, such as di-2-ethylhexyl phthalate (DEHP), DnBP, DMP, and DEP, as well as several OPFRs, such as TBEP and TBP, which were associated with a higher risk of GDM. The obtained results were analyzed using weighted quantile sum regression (WQS) and Bayesian kernel machine regression (BKMR). The WQS model showed that the combination of PAEs and OPFRs was positively associated with the risk of GDM, and importantly, the BKMR analysis confirmed these results. The association of exposure to the combination of substances with GDM varied between the 55th and 75th percentiles. It should also be noted that the article has a certain limitation. The researchers measured compounds that are largely metabolized in the urine. Therefore, the results should be supported by additional studies that take into account the identified metabolites of these compounds (Lang et al., 2024).

Phthalates may also contribute to type 2 diabetes (T2DM), which is associated with insulin resistance and hyperglycemia. Over time, a growing number of studies have demonstrated a significant impact of EDCs on the incidence of this condition. For example, a group of researchers obtained data from the NHANES website in 2023 from 1,042 adults who had complete information on the presence of DEHP metabolites and T2DM. Of all the participants, only 104 had diabetes, while 938 were healthy. As part of the study, the concentrations of several phthalate metabolites were measured and conclusions were drawn. Higher levels were observed for the compounds mono- (2-ethyl-5-hydroxyhexyl) phthalate (MEHHP), mono-2-ethyl-5-carboxypentyl phthalate (MECPP), mono- (2-ethyl-5-oxohexyl) phthalate (MEOHP), and ΣDEHP. A ΣDEHP variable was based on MEOHP and MEHHP, which were significantly associated with the risk of T2DM, although the correlation was nonlinear. Individuals with T2DM were also found to be older and had higher BMI, systolic blood pressure, waist circumference, total cholesterol, fasting glucose, fasting insulin, ΣDEHP, and the metabolites MEHHP, MECPP, and MEOHP. Researchers suggest that exposure to DEHP metabolites contributes to metabolic disturbances caused by insulin resistance, altered PPAR signaling pathways, and fatty acid degradation (Xue et al., 2023).

There is also a 2022 study by Todd et al., which examined the exposure of phthalate metabolites to the risk of GDM in each trimester of pregnancy. A positive correlation was found in the second trimester, where MiBP concentrations were significantly higher and associated with increased impaired glucose tolerance. In the same trimester, increased concentrations of mono-3-hydroxybutyl phthalate (MHBP) were also observed, associated with an increased risk of GDM. The only difference was that the effect of this compound increased non-monotonically compared to MiBP (James-Todd et al., 2022).

In 2022, researchers Liang et al. identified 11 phthalate metabolites in a group of 200 pregnant women from whom morning urine was collected. Statistically significant differences were found for the compounds MEHP and MECPP, which were higher in the group of women with GDM than in the control group. Additionally, Spearman correlation analysis was performed, confirming the association between phthalate metabolites and the risk of GDM. The results showed a correlation between blood glucose levels and the concentrations of MEOHP, MECPP, and MiBP. Fasting insulin levels correlated with the concentrations of the metabolites MEP, MiBP, MMP, and MECPP, as did the insulin resistance index with the concentrations of MiBP, MMP, and MECPP. According to the researchers, factors such as the pregnant woman’s age >35 years, pre-pregnancy BMI, and gestational age >28 weeks contributed to the increased risk of GDM (Liang et al., 2022).

Although most studies confirm the effect of phthalates on the risk of GDM, some results do not provide clear evidence of this correlation. For example, a study conducted on pregnant Latina women examined urine for 11 different phthalate metabolites and found higher MEP concentrations and higher mean concentrations, including MiBP and ΣDEHP, in mothers who were obese before pregnancy. Importantly, however, MEP concentrations only slightly increased the risk of excessive weight gain during pregnancy, while other metabolites increased the risk of higher pre-pregnancy BMI. Compared to other studies, this study found no association between phthalates and glycemia in pregnant women (Zukin et al., 2021; International Diabetes Federation).

In 2022, a research project conducted at the University of Medical Sciences in Isfahan was published. The aim was to investigate the relationship between individual phthalate metabolites and T1DM in children. The study involved 294 children, 147 of whom were recently diagnosed with T1DM and 147 controls. Four phthalate metabolites were measured in urine: MEOHP, MEHHP, MBzP, and MnBP. The results showed significantly higher concentrations of all these compounds in the study group compared to the control group. Analyses showed that only high MEOHP concentrations were statistically significant and may increase the risk of developing T1DM in children. Other metabolites were not statistically significant. This study was conducted as a case-control design (Hashemipour et al., 2022). To definitively determine the effect of phthalates on the development of T1DM, newer and more extensive studies involving a larger number of individuals are needed. At this time, we can only speculate about their effects on the body and whether this is related to the development of T1DM in children.

These findings, which are also summarized in Table 1, suggest that phthalates may play an important role in the development of diabetes.

**TABLE 1:** Epidemiological studies assessing associations between phthalate exposure and metabolic outcomes.

Author, year	Country/Population	Study design	Sample size	Phthalate(s)/Metabolite(s) assessed	Biological matrix	Exposure assessment	Metabolic outcome(s)	Main findings
(Xue et al., 2023)	China/United States of America	Bioinformatic Analysis Datasets from the National Health and Nutrition Survey (NHANES)	N = 1,042 adults	DEHP metabolites: MBzP, MEOHP, MiBP, MEHHP, MEP, MC1P, MnBP, MECPP, MCOP, MCNP	Urine	High-performance liquid chromatography-electrospray ionization-tandem mass spectrometry	Individuals with T2DM, beside higher level of compunds had higher: BMI level, systolic blood pressure, waist circumference, total cholesterol, fasting glucose, fasting insulin	Higher levels were observed for the compounds MEHHP, MECP, MEOHP, and ΣDEHP. Exposure to DEHP metabolites contributes to metabolic disturbances caused by insulin resistance, altered PPAR signaling pathways, and fatty acid degradation
(Lang et al., 2024)	Liuzhou, Guangxi, China; pregnant women aged 20–45 years, enrolled at 10–14 gestational weeks	Nested case-control study within a prospective birth cohort	N = 165 65 GDM cases; 100 controls	Parent PAEs: DEHP, DnBP, DMP, DEP (metabolites not assessed); additionally, 7 OPFRs were assessed as co-exposures	First-trimester urine	Gas chromatography-tandem triple quadrupole mass spectrometry (GC-MS) concentrations corrected for urinary creatinine	Gestational diabetes mellitus (GDM), diagnosed by 75-g OGTT	DBP, DEHP, DEP and DMP were positively associated with GDM in the confounder-adjusted model), whereas BBP was not. Individual PAE associations were attenuated after adjustment for co-exposures. The overall OPFR–PAE mixture was positively associated with GDM, although the mixture effect was mainly driven by OPFRs
(James-Todd et al., 2022)	United States of America; pregnant women participating in the LIFECODES pregnancy cohort, data collected 2009–2017	Prospective cohort analysis	N = 606 normoglicemia = 136; IGT = 296; gdm = 174	19 biomarkers of phthalates and replacements: MEP, MBP, MHBP, MiBP, MHiBP, MCPP, MBzP, MEHP, MEHHP, MEOHP, MECPP, MCNP, MCOP, MNP, MONP, MECPTP, MEHHTP and DINCH metabolites MHiNCH and MCOCH; metabolites also evaluated as chemical mixtures	Urine	Spot urine samples collected up to four times during pregnancy; 1st- and 2nd-trimester measurements obtained before glucose testing were used prospectively; solid-phase extraction followed by HPLC and isotope-dilution tandem MS; specific-gravity correction; PCA used for mixture analyses	Maternal glucose intolerance classified as normoglycemia, IGT, or GDM using 50-g 1-h glucose challenge followed, when indicated, by 100-g 3-h OGTT	Associations were trimester- and metabolite-specific. Higher 1st-trimester MCPP was associated with lower odds of and IGT. Higher 2nd-trimester MiBP was associated with increased odds of IGT. Second-trimester MHBP showed a non-monotonic positive association with GDM. A 2nd-trimester mixture dominated by dibutyl-phthalate metabolites was associated with increased IGT
(Liang et al., 2022)	Guangzhou, China; pregnant women ≥18 years with singleton pregnancies	Cross-sectional study/case-control comparison of women with and without GDM	N = 200; GDM = 100; Controls = 100	11 metabolites: MMP, MEP, MBP, MCHP, MBzP, MiBP, MEHP, MEOHP, MECPP, MOP, MiNP	Morning urine	Urinary metabolites measured after enzymatic deconjugation using LC–MS/MS/UPLC–MS/MS; morning/clinic urine samples; no creatinine correction	GDM; fasting glucose; fasting insulin; insulin resistance index	glucose; fasting insulin; insulin resistance index MEHP and MECPP concentrations were higher in women with GDM. After multivariable adjustment, MEHP remained positively associated with, whereas MECPP did not. MiBP, MEOHP and MECPP correlated positively with fasting glucose; MMP, MEP, MiBP and MECPP with fasting insulin; and MMP, MiBP and MECPP with insulin resistance
(Zukin et al., 2021)	United States of America; pregnant Latina women from the CHAMACOS cohort, predominantly from a low-income agricultural community	Prospective birth cohort study	N = 415; GDM = 31; IGT = 49; excessive GWG = 223	11 metabolites: MEP, MBP, MiBP, MBzP, MEHP, MEHHP, MEOHP, MECPP, MCPP, MCOP, MCNP; four DEHP metabolites combined as ΣDEHP	Urine	Two spot urine samples collected at mean 14.2 and 26.9 gestational weeks; solid-phase extraction and isotope-dilution HPLC–ESI–MS/MS; urinary dilution corrected using specific gravity; average of the two measurements used in primary analyses	GDM, impaired glucose tolerance (IGT), GCT plasma glucose concentration, excessive gestational weight gain (GWG), pre-pregnancy BMI	No significant associations were observed between any phthalate metabolite and GDM, IGT, or plasma glucose concentrations. Higher MEP was weakly associated with excessive GWG. Women with higher pre-pregnancy BMI had higher concentrations of several metabolites; significant differences were observed for MiBP, MBzP, MCOP and ΣDEHP.

### The role of microbiota in phthalate exposure and links to obesity, diet, and diabetes

The function of the intestinal microbiota has a significant impact on the absorption and metabolism of nutrients and on the overall functioning of the digestive, nervous, and immune systems. They also mitigate disruptions to the intestinal barrier by binding to contaminants to accelerate their excretion and limit absorption. Due to the fact that some phthalates can enter living organisms through oral exposure, researchers began to wonder what role the microbiota plays in this whole process. Environmental pollutants disrupt the homeostasis of the gut microbiome, affecting many key processes. By examining the composition of the microbiome, it is possible to assess the toxicity of phthalates, which exert a selective inhibitory effect on the gut microbiome. *Lactobacillus* populations, known for their beneficial anti-inflammatory effects, are displaced by other bacterial populations. Lan et al., examining the synergistic effects of microplastics and DEHP on enterohepatic toxicity, found an increased inflammatory response coupled with oxidative stress and disruption of the gut microbiota. Transplantation of fecal microbiota from treated mice to healthy mice resulted in similar toxic effects. Their findings suggest a significant role for microbiota in the mechanism of phthalate- and microplastic-induced toxicity. However, no specific microbiome signature has been validated as a biomarker of phthalate toxicity (Lan et al., 2026; Liu et al., 2026; Zarezadeh et al., 2026).

According to the literature, the gut microbiota of healthy people is dominated by two types of bacteria: *Bacteroidetes* and *Firmicutes* (Singh et al., 2022). An elevated *Firmicutes*-to-*Bacteroidetes* ratio is very often considered a trait correlated with obesity, suggesting that this ratio may serve as a potential biomarker. Over the years, however, studies have emerged in which no significant differences in this parameter were observed between individuals of normal weight and obese individuals. Several studies have reported a decrease in this ratio in obese humans and animals. This has sparked controversy regarding the use of the *Firmicutes*/*Bacteroidetes* ratio as a characteristic feature of obesity (Singh et al., 2022; Cheng et al., 2022; Magne et al., 2020). Nevertheless, this parameter has been included in studies evaluating the effect of exposure to selected phthalates on the gut microbiota. In most cases, an increase in the abundance of *Firmicutes* (Li et al., 2025; Fu et al., 2021; Wang et al., 2019; Xiong et al., 2020; Wang et al., 2020) and a decrease in the abundance of *Bacteroidetes* were observed; however, in some studies, the ratio of these bacteria remained unchanged or decreased (Hu et al., 2016; Shum et al., 2023; Chiu et al., 2021).

Research conducted by Sheng-long Cao et al. showed that exposure to DEHP can lead to dysregulation of lipid metabolism by altering the composition of the gut microbiome. In particular, an increased abundance of Prevotellaceae, a group of anaerobic Gram-negative bacteria belonging to the Bacteroidetes phylum, was positively correlated with elevated triglyceride levels in mouse adipose tissue. Bacteria from this family are associated with lipid disorders, and their abundance in the microbiome is observed in obese individuals (Cao et al., 2025; Zhao et al., 2021).

Butyrate plays a significant role in maintaining intestinal homeostasis. It is a microbial metabolite used by colon epithelial cells as an energy source. The literature indicates its beneficial effects on the nervous system and its role in regulating the immune response. In studies conducted by Ming Lei et al., exposure to DEHP correlated with a decrease in butyrate in an *in vitro* gut microbiota model (Corrêa-Oliveira et al., 2016; Chiu et al., 2020).

Evidence presented by Chakaroun et al. further supports a relationship between the gut microbiome and metabolic obesity rather than a simple unidirectional causal pathway. They have analyzed 1,408 deeply phenotyped individuals and developed a metabolome-informed BMI (metBMI) that identified metabolic dysfunction independently of conventional BMI. They found a relationship between high metBMI and greater visceral adiposity, insulin resistance, hyperinsulinemia, systemic inflammation, and reduced gut microbial gene richness. The gut microbiomes of those individuals had reduced ability to produce butyrate. Their findings suggest contribution of microbiota to metabolic dysfunction and responsiveness to host metabolic and dietary alterations. This is further supported by Asnicar et al. who analyzed 1,203 gut metagenomes from 1,098 individuals. Diet is a strong determinant of the microbiota, and the microbiota is strongly associated with obesity and metabolic response (Asnicar et al., 2021; Chakaroun et al., 2026).

Despite the limitations of *in vitro* studies, similar results are presented in a study by Gang Wang et al., where a correlation was observed in the feces of Sprague-Dawley (SD) rats between a decrease in butyrate concentration and DEHP exposure. Additionally, these animals exhibited the most significant weight gain compared to other groups, which may result not only from intestinal dysbiosis but also from the potential role of butyrate in preventing obesity (Wang et al., 2020).

In a cross-cohort meta-analysis study by Mei et al., 8,117 metagenomes from multiple populations were analyzed to assess, among other things, the overall microbial community structure and strain-specific functions in an international collection of population-based microbiome studies of participants with type 2 diabetes, prediabetes, and normoglycemic status. While this does not provide conclusive evidence, it provides a basis for further research into the pathogenesis of T2D and the involvement of dysbiosis of the gut microbiome (Mei et al., 2024).

Multi-omics studies indicate that the metabolic consequences of gut microbiota alterations may be mediated through changes in circulating metabolites rather than through taxonomic shifts alone. Wu et al. analyzed 1,167 individuals spanning normal glucose tolerance, prediabetes, and treatment-naive type 2 diabetes, identifying 143 circulating metabolites that were associated with the gut microbiome. The study revealed extensive microbiome-metabolome interactions involving metabolites linked to amino acid, lipid, xenobiotic, and benzoate metabolism. The findings in this study support the concept of a modifiable diet-microbiome- metabolome axis contributing to glucose regulation (Wu et al., 2025).

Gut microbiota occupies a dual position in the relationship between phthalate exposure and metabolic dysfunction, acting both as a downstream biological target of exposure and as a potential mediator of adverse metabolic effects. Experimental studies have consistently demonstrated that exposure to phthalates, such as DEP, DBP, DEHP, and MEHP, can alter gut microbial composition and function, indicating that dysbiosis represents a direct biological consequence of exposure. Mechanistic evidence suggests that these microbial alterations may subsequently contribute to metabolic disturbances through impairment of intestinal barrier integrity, increased translocation of bacterial lipopolysaccharide, activation of inflammatory pathways, alterations in microbial metabolites, and disruption of liver and adipose signaling. Xiong et al. demonstrated that DBP-induced dysbiosis was accompanied by impaired intestinal barrier function, increased circulating LPS, activation of hepatic TLR4/NF-κB signaling, and disturbances in lipid metabolism, whereas Cao et al. linked DEHP-induced microbial alterations with impaired thyroid hormone signaling, adipose lipid accumulation, and an obesogenic phenotype through the gut-adipose tissue axis. However, the gut microbiota should currently be regarded as a potential mediator rather than a definitively established causal intermediate between phthalate exposure and obesity or diabetes. Its potential role as an effect modifier of host susceptibility is biologically plausible, particularly in the context of dietary influences, whereas the use of microbiota profiles as specific biomarkers of phthalate exposure remains insufficiently validated (Xiong et al., 2020; Hu et al., 2016; Shum et al., 2023; Cao et al., 2025).

Moreover, there is increasing talk of probiotics, which are intended to prevent the destruction of intestinal microbiota by environmental pollutants. They may compensate for the effects of contamination with phthalates, among others, by improving intestinal barrier function and enhancing the metabolism of intestinal mucosal cells. Probiotics and prebiotics enhance antioxidant and anti-inflammatory effects. The definitions of probiotics, prebiotics, and synbiotics are provided by the International Scientific Association for Probiotics and Prebiotics (ISAPP) (Zarezadeh et al., 2026).

Kefir and yogurt are sources of probiotics. They contain yeast and species of *Lactococcus*, *Leuconostoc*, and *Acetobacter*. They are classified as functional foods, meaning they provide nutritional support while also improving physiological functions and reducing the risk of disease. They also help reduce the number of bacteria with a negative impact, such as *Bacteroidetes*, and increase the number of beneficial bacteria, such as *Firmicutes* and *Actinobacteria*. The ratio of Firmicutes to Bacteroidetes bacteria can provide additional information about the course of diseases. However, the direction of these alterations is inconsistent across studies. An increased ratio correlates with chronic diseases, while a decreased ratio is associated with improved metabolic health. Such diseases include metabolic syndrome, fatty liver disease, and obesity (Wang et al., 2020).

Interesting research was conducted by Jia et al. They found that consuming probiotics during pregnancy can increase the gut microbiome and reduce infant weight. Probiotics, in addition to bacterial cultures that have health benefits, also contain high-quality nutrients, protein, vitamins, and phospholipids. In another study, pregnant women diagnosed with gestational diabetes who included probiotics in their diets demonstrated reduced glucose, insulin, and homeostatic model assessment of insulin resistance (HOMA-IR) levels. The same phenomenon was observed with synbiotic supplementation (Jia et al., 2026; Rosenfeld, 2024).

Human studies provide increasing evidence of internal exposure to phthalates and their association with microbial composition and function. Infants’ and children’s urine analyzed in other studies confirmed reduced diversity of microbes in association with exposure to phthalates. However, they also have their limitations. They remain largely cross-sectional and do not establish whether microbiota alterations precede metabolic dysfunction, mediate phthalate toxicity, or arise as secondary consequences of differences in diet, metabolic status, or other co-exposures. On the other hand, controlled animal studies provide stronger evidence on biological plausibility. Chronic low-dose DEHP exposure is associated with high-fat diet induced obesity, while at the same time remodeling gut microbial communities, altering PPARγ signaling, disturbing thyroid functions, and lipid accumulation in adipose tissue (Liu et al., 2023; Cao et al., 2025; Jia et al., 2026; Davias et al., 2024; Zhang et al., 2026; Wei et al., 2022; Wu et al., 2026).

Reviewed literature used to assess microbiota’s possible role in phthalate exposure and its links to obesity, diet, and diabetes are presented in Table 2.

**TABLE 2:** Studies investigating the effects of phthalates on gut microbiota.

Author, year, study model/Population	Phthalate(s) investigated	Exposure dose/Concentration; exposure duration	Main microbiota alterations	Changes in microbial metabolites/Intestinal function	Metabolic effects	Proposed mechanism	Main limitations
(Hu et al., 2016) Female Sprague-Dawley rats; postnatal exposure	DEP; additionally MPB, TCS and mixture	DEP 0.1735 mg/kg bw; environmentally relevant low-dose exposure PND 1-181; feces assessed at PND 62 and 181	At PND 62 ↑ Bacteroidetes/Prevotella ↓ Firmicutes/Bacilli ↑ Elusimicrobia in DEP and MPB groups; microbiota differences largely disappeared by adulthood	Not characterized	Small reduction in body weight during adolescence, particularly after DEP and MPB exposure	Early-life low-dose environmental exposure may transiently modify the gut microbiota during a susceptible developmental window	Only female rats; microbiota effects mainly transient; DEP was only one component of a broader environmental-chemical experiment; downstream metabolic causality not demonstrated
(Wang et al., 2019) Male C57BL/6 mice exposed during puberty; normal or high-fat diet	MEHP	0.05 mg/kg bw; 4 weeks	↑ Firmicutes ↓ Verrucomicrobia ↑Intestinimonas, Holdemanella, Coprobacter and Parasutterella ↓Akkermansia, Tannerella and Alloprevotella	Several bacterial taxa correlated with cholesterol burden	↑ fat mass, total cholesterol, HDL-C and LDL-C under HFD; adipocyte hypertrophy and cholesterol overload; altered hepatic cholesterol deposition/transport	MEHP-induced microbiota alterations, particularly in combination with HFD, may contribute to disrupted cholesterol homeostasis and adipose/liver dysfunction	Animal model; dietary interaction is substantial; single low MEHP dose; association between microbial taxa and lipid phenotype does not by itself establish microbiota-mediated causality
(Wang et al., 2020) Male prepubertal SD rats, Wistar rats, BALB/c mice and C57BL/6J mice	DEHP	0; 300; 1,000; 3,000 mg/kg/day in drinking water; 30 days	Strongly species/strain-dependent effects. SD rats showed the greatest dysbiosis, including ↑ Firmicutes/Bacteroidetes ratio and ↑ Proteobacteria	↓ butyrate ↑ potential pathogenic bacteria and microbial functions associated with metabolic/inflammatory diseases	SD rats showed greatest body-weight gain, organ injury and Th1 inflammatory response	DEHP-induced dysbiosis and reduced butyrate production may contribute to inter- strain differences in metabolic toxicity and inflammation	Very high experimental doses; marked species/strain specificity; findings cannot be directly extrapolated to humans; microbiota causality not directly tested
(Xiong et al., 2020) 6-week-old C57BL/6J mice	DBP	0.1 or 1 mg/kg by oral gavage; 10 weeks	DBP disturbed gut microbiota homeostasis; significant taxonomic alterations were evident, particularly at 0.1 mg/kg	↓Intestinal mucus secretion and impaired barrier function; **↑** serum LPS	↑ body weight ↑serum/hepatic TG and total cholesterol; increased hepatic lipogenic gene expression and inflammation	Dysbiosis → impaired intestinal barrier → LPS translocation → TLR4/NF-κB activation→ IL-1β, IL-6, TNF-α → hepatic inflammation and disturbed lipid metabolism ↓PPARγ	Mouse model; only two DBP doses; microbiota changes were associated with, rather than definitively proven to cause, metabolic effects
(Fu et al., 2021) Female ICR mice	DEHP	500 and 1,500 mg/kg bw/day 30 days	Significant restructuring of gut bacterial communities ↑ Firmicutes, Akkermansia, Turicibacter, Romboutsia, Verrucomicrobiales ↓Bacteroidetes, Actinobacteria, Epsilonbacteraeota, *Bacteroides*/Bacteroidaceae Clear separation of DEHP groups from controls	Altered fecal metabolome, including SCFA profiles Changes in phenylalanine metabolism, steroid biosynthesis, purine/pyrimidine and other pathways	Systemic inflammation, ovarian oxidative stress, reduced estradiol, ovarian histological abnormalities and reproductive toxicity	DEHP → dysbiosis + altered microbial metabolites/SCFAs → systemic inflammation and oxidative stress → reproductive toxicity	Very high DEHP doses; reproductive rather than primarily metabolic endpoint; no direct microbiota-transfer experiment demonstrating causality
(Chiu et al., 2021); pregnant mice	DEHP	20 μg/kg/day, environmentally relevant dose, orally 10 days during pregnancy	↑ α-diversity, altered β-diversity ↑ Bacteroidetes ↓ Firmicutes and Deferribacteres; alterations involving Deferribacteraceae, Lachnospiraceae and Mucispirillum	14 microbial functional pathways significantly altered	No significant effect on fertility or measured birth outcomes	Gestational DEHP exposure can induce persistent remodeling of maternal cecal microbiota even without overt reproductive phenotype	Animal model; one exposure dose; significant pregnancy outcomes did not accompany microbiota alterations; metabolic endpoints were limited
(Shum et al., 2023) *In vitro* human fecal microbiota fermentation; microbial extracts subsequently tested on HT-29 enterocytes and RAW264.7 macrophages	DEHP, DINP	DEHP 1.05; 10.5; 105 μg/mL; DINP 0.168; 1.68; 16.8 μg/mL 24 h anaerobic fermentation	DEHP altered microbial composition and diversity and reduced the Firmicutes/Bacteroidetes ratio High-dose DINP promoted Proteobacteria and altered multiple genera	Microbiota-derived luminal extracts altered epithelial tight/adherens-junction gene expression; effects on CLDN2, CLDN4, OCLN, JAMA, CDH1, TJP1; suppression of anti-inflammatory macrophage genes including IL10/IL10R2/IRF4 with DINP	No direct whole-body metabolic phenotype; evidence of impaired epithelial integrity and altered immune homeostasis	Phthalate-induced dysbiosis may generate microbial products that promote leaky gut and intestinal inflammation, providing a plausible link to systemic metabolic inflammation	*In vitro* model, relatively high concentrationsshort exposure, no intact host physiology; cannot directly infer clinical metabolic disease
(Cao et al., 2025) Male SAMP8 senescence-accelerated mice; aging model	DEHP	0; 0.2 or 200 mg/kg/day orally 5 weeks	DEHP disrupted α- and β-diversity; high dose reduced microbial richness; notably ↑ Prevotellaceae_UCG-001, which correlated positively with TG in eWAT	Gut–fat axis alterations; microbial dysbiosis accompanied by impaired thyroid-hormone signaling	↑ body weight and adipocyte hypertrophy, especially at 0.2 mg/kg/day; impaired energy expenditure and BAT thermogenesis; lipid accumulation in eWAT; ↓ T3/T4 and impaired DIO/TR signaling	DEHP → microbiota dysbiosis + impaired thyroid hormone signaling → disrupted gut–fat axis → adipose lipid accumulation and obesogenic phenotype	Aging mouse model; substantial difference between low and high doses; relatively short exposure; correlation between specific taxa and adipose lipids does not prove microbial causation

## Summary

The progress of civilization is associated with increased human exposure to harmful substances, including microplastics and phthalates, which significantly impact the metabolic health of the population, and contact with them is practically unavoidable.

Current regulations restrict the use of the most toxic phthalates, but their widespread presence still poses a significant public health challenge and requires further research and preventive measures. A balanced diet is crucial for maintaining health and preventing and treating metabolic diseases, but its effectiveness can be undermined by the presence of chemical contaminants in food and water, making the diet a significant source of exposure to these compounds.

Completely eliminating contact with phthalates is currently impossible; further research, more effective quality control of food and packaging, and increasing consumer awareness of potential hazards are necessary. Plastic recycling, while environmentally beneficial, can increase the risk of phthalates in final products if appropriate safety standards are not met.

The presence of phthalates in the aquatic and soil environment poses a risk of their entry into the food chain, endangering not only the health of humans but also that of farmed and wild animals. Pregnant women, infants, and children are particularly vulnerable to the negative effects of phthalate exposure, as these compounds can cross the placenta and affect fetal development and lipid metabolism.

Obesity should not be viewed solely as a consequence of excessive energy intake, but also as a consequence of long-term environmental exposure to substances that disrupt metabolism. Further research into the mechanisms of action of obesogens and preventive measures limiting exposure to EDCs is necessary to more effectively prevent obesity and metabolic diseases.

Phthalates can disrupt glucose homeostasis through multiple mechanisms, including impaired pancreatic β-cell function, induction of insulin resistance, oxidative stress, and alterations in PPAR signaling pathways.

Gut microbiota dysbiosis may be an important mechanism linking phthalate exposure with metabolic disturbances, because of their abilities to alter microbial composition, reduce butyrate production, promote inflammation and oxidative stress, and contribute to lipid metabolism disorders. Further research is required to confirm the effectiveness in preventing or reducing phthalate-related metabolic dysfunction by probiotics, prebiotics, and synbiotics. The gut microbiota should be considered both a downstream biological target of phthalate exposure and a potential mediator of phthalate-associated metabolic effects. Its role as an effect modifier remains plausible but insufficiently demonstrated, whereas its application as a biomarker is still exploratory.

Current evidence suggests that phthalates may contribute to the development of diabetes, particularly gestational diabetes and type 2 diabetes, underscoring the need for larger, longitudinal studies and the development of preventive strategies to reduce exposure to environmental chemicals. Overall, current evidence suggests that the gut microbiota functions both as a downstream biological target of phthalate exposure and as a potential mediator of metabolic toxicity. Experimental studies indicate that phthalate-induced dysbiosis may contribute to intestinal barrier dysfunction, altered microbial metabolite production, inflammatory signaling, and disruption of gut–liver and gut–adipose communication. However, causal mediation has not been formally demonstrated in most studies, particularly in humans. The role of the microbiota as an effect modifier appears plausible and context-dependent, particularly in relation to diet, metabolic status, sex and developmental stage, whereas its application as a biomarker of phthalate exposure remains insufficiently validated (Xiong et al., 2020; Hu et al., 2016; Shum et al., 2023; Cao et al., 2025).
